# Psychotherapists’ emotional reactions to patients’ personality trait in personality disorder treatment settings: an exploratory study

**DOI:** 10.1186/s40359-021-00580-z

**Published:** 2021-05-06

**Authors:** Cesare Cavalera, Annalisa Boldrini, Alessia Antonella Merelli, Edoardo Squillari, Pierluigi Politi, Francesco Pagnini, Osmano Oasi

**Affiliations:** 1 Department of Psychology, Università Cattolica del Sacro Cuore, Milan, Italy; 2 Department of Mental Health and Addiction, ASST Papa Giovanni XXIII, Bergamo, Italy; 3 Department of Mental Health and Addiction, ASST Ovest Milanese, Milan, Italy; 4 Department of Brain and Behavioral Sciences, University of Pavia, Pavia, Italy; 5 Department of Psychology, Harvard University, Cambridge, MA USA

**Keywords:** Emotion in therapy, Personality disorders, Alliance

## Abstract

**Background:**

Therapist’s emotional reactions toward patients in clinical facilities are a key concept in the treatment of personality disorders. Considering only clinical settings specialized in treatment of personality pathology the present paper aimed at: (1) assessing any direct relationship between patient symptom severity and therapist emotional response; (2) exploring patients’ functioning configurations that can be associated with specific therapist reactions (3) investigating whether these relationships remains significant when accounting for other setting variables related to patients or therapist.

**Methods:**

The present study included 43 outpatients with personality disorders who underwent a psychotherapy treatment in two Italian facilities dedicated to outpatients with personality disorders and their 19 psychotherapists. The Symptom Checklist-90-Revised (SCL-90R) was used to explore clinical severity condition. Psychotherapists completed the Therapist Response Questionnaire (TRQ) to identify pattern of therapists’ response and the Shedler-Westen Assessment Procedure-200 (SWAP-200) in order to assess personality traits of the patients.

**Results:**

No significant relationship between the clinical severity of the symptoms and the therapist’ responses was found. Even when controlled for clinical severity condition, duration of the treatment, age and educational level of the patient or years of therapist experience, most of SWAP-200 traits appeared to be significant predictors of therapist’ emotional responses.

**Conclusions:**

The present study confirms the value of therapists’ emotional response as a useful tool in understanding psychological processes related to clinical practice highlighting its context-dependent dimension.

**Supplementary Information:**

The online version contains supplementary material available at 10.1186/s40359-021-00580-z.

## Introduction

Treating people with personality disorders (PD), in particular with borderline personality disorder, can trigger intensive emotional reactions in the psychotherapist [1]. Recognizing and understanding the therapist emotional reactions has crucial implications for treatment, not only for the on-going psychotherapy in outpatient facilities but also for briefer encounters in emergency departments [2].

The influence of specific personality syndromes on the patient-therapist relationship has already received attention from the scientific community. Rossberg and colleagues [3, 4] documented that patients with cluster A and B personality disorders were related to more negative and less positive therapist emotional responses than those with cluster C personality disorders, and patients who dropped out of treatment evoked more negative countertransference reactions than patients who completed the treatment. Negative emotional reactions when dealing with cluster B personality disorders, which seems to be related to more mixed and negative responses in their therapists than clusters A and C personality disorders, were also found by Colli and colleagues [5] and by Tanzilli and colleagues [6]. The available evidence confirms that patients with PD, especially borderline disorders, tend to be associated with a strong emotional reaction in the therapist. However, this reaction was found in large samples of psychiatrists and clinical psychologists that explored cases taken from their private practice psychotherapies and not exclusively from centers specialized in the treatment of personality disorders [5, 6].

Level of interpersonal functioning and personality style seem to have a stronger correlation with countertransference feelings than with a patient’s general level of functioning or with his or her level of symptoms severity [7, 8], Dimaggio and colleagues’ [9] evidenced that symptomatic condition appears to be related to the outcome of the treatments of patients with personality disorders. This may be true for some personality disorders (i.e., schizotypal, borderline, histrionic, and avoidant) that showed that the symptomatology partially mediates the relationship between their personality disorders and their therapists’ emotional responses. In these cases, the severity of clinical condition seems to be related with stronger degree of negative emotional responses [8]. As the different therapeutic approaches and other variables of the therapist (as gender, age, profession, and experience) seem to be not significantly related to countertransference reactions [8], the type of setting analysed could be a key element that could explain this phenomenon.

All these previous studies have primarily explored these associations in clinical settings, either public or private, without a specific focus on PDs. Therapists that work in facilities specialised in PD may be used to deal with patients with these mental disorders and are more likely to manage emotional reactions beyond the patients’ level of severity. This may be due to continue opportunities of experiential learning, regular clinical supervision and reflective practice that are focused on these specific kind of patients and that can help in the recognition of the emotional impact that such individuals have on their therapists [10].

Finally, how patients’ personality traits relate to other variables in determining countertransference reactions is still a subject of wide scientific debate. More specifically, little research has examined which characteristics of a patient or of the clinician (e.g., age, gender) are most likely to evoke negative reactions in the therapist [11]. Although Lingiardi and colleagues [8] suggest that descriptive information related to the psychotherapists and their patients don’t have a key role in determining therapists’ emotional reactions, Liebman and Burnette [11] showed a number of client- and clinician- level factors that impact on countertransference reactions. A better understanding of the relationship between these variables can help to better identify therapists’ emotional reactions and the way they may impact treatment decisions.

Therefore, the goal of the present study is to explore the therapist’s emotional reactions toward patients with PDs involving clinical settings specialized in treatment of personality pathology (particularly borderline traits). We address three specific research questions: (1) Is there a relationship between patients’ symptom severity and therapist emotional response? (2) Are there patients’ functioning configurations that can be associated with specific therapist reactions? (3) Do correlations between countertransference and patient personality functioning remain significant also when accounting for variables such as patient or therapist characteristics (mean age, years of therapeutic experience)?

Given frequent compromised clinical conditions shown by patients treated in mental health facilities, we hypothesize that symptom severity won’t be related with therapist emotional responses that are use to work with such patients with PD. Conversely with previous work [5, 6], we can prudentially expect that borderline traits (SWAP borderline PD score) will not be related with strong emotional reaction in the therapists. This can be hypothesized because borderline patients are commonly treated in the present therapeutic context. Differently, we can expect that disorders that are less commonly treated in these kind of facilities such as A or C clusters may be related with difficult-to-manage emotions. Moreover, following Lingiardi and colleagues findings [8] it can be hypothesized that countertransference reactions won’t be accounted for psychotherapists and patients characteristics or other setting variables (e.g. age, duration of the treatment).

## Methods

### Patient characteristics

49 patients were asked to enter the study, but 6 patients refused (12.24 %). The final sample is composed by 43 outpatients with PDs (Female N = 26, 60.5 %; Male N = 17, 39.5 %) who underwent a psychotherapy treatment (at least six sessions). The therapy was administered in two Italian facilities dedicated to outpatients with personality disorders treatment: *Centro Interdipartimentale per la Ricerca sui Disturbi di Personalità* [Inter-departmental center for personality disorder research], located in Pavia and *Casa di Howl* [Howl’s house] located in Genoa.

## Therapists

19 therapists of the two involved centers accepted to join the study and proposed the present research to eligible patients. The recruitment started on April 2017 and ended on September 2018. Once obtained the consent from therapists and patients, the questionnaires were delivered. Ethical approval was granted by the ethical committees of the Pavia Inter-departmental center for personality disorder research.

## Assessment measures

Descriptive information related to the psychotherapists and their patients were collected in order to have details about the different clinical situations involved (age, years of study, number of hospitalizations, years of therapist experience and duration of the treatment).

The *Shedler-Westen Assessment Procedure-200* (SWAP-200) [12, 13] was used to assess the personality of the patients. This Q-sort instrument includes 200 statements describing several aspects of personality, each of which may describe a given patient well, somewhat, or not at all. The clinician ranks these statements into eight categories from those that are most descriptive (assigned value of 7) to those that are not descriptive (assigned a value of 0). This instrument provides a personality assessment expressed by a final rating divided in 11 Personality Disorders factors (PD T-scores) and 13 Q sort factors (Q scores). While PD T-scores are related with DSM-IV personality disorder as explained in Axis II diagnosis, Q sort factors scores for an alternative set of personality syndromes often seen in clinical practice that addresses limitations of the DSM-IV diagnostic system. Indeed, this procedure is “bottom-up”: it means the clinician tries to compare his patient with the prototype of a specific personality disorder and to define how his patient is near to this prototype. In this way, “Q-factor analysis identifies groups of similar people who share a common syndrome” [13]—not groups of diseases. The Italian version of the SWAP was used [14]. The present instrument has been widely used in process and outcome research [e.g., 15] as well as on group studies with a variety of clinical populations and measures [16]. Previous findings has evidenced that the present instrument is a valid and reliable tool that can facilitate diagnosis process: reliability of SWAP–200 personality descriptions has ranged from 0.75 to 0.89 (Marin-Avellan, McGauley, Campbell, and Fonagy 2005; Shedler and Westen 1998; Westen and Muderrisoglu 2003). Moreover, interrater reliability of SWAP diagnostic scales assessed by independent clinicians and the treating clinicians averaged greater than 0.80 for all SWAP diagnostic scales [17].

The *Symptom Checklist-90 Revised*. The SCL-90 R [18] is a widely used self-report assessing 90 psychiatric symptoms on a 5-point Likert scale, ranging from 0 (not at all) to 4 (extremely). SCL-90-R explores how much the patient had “been distressed” by the symptom within the past seven days. The Global Severity Index (GSI) score, which is the mean rating across all 90 items that summarizes the client’s general psychiatric symptom severity, was used for the present study. The present scale has previously shown a good level of validity and reliability with a Cronbach alpha coefficient higher than 0.90 [19].

*The Therapist Response Questionnaire* (TRQ) [20, 21] is a clinician questionnaire designed to explore the emotional responses of psychotherapists to their patients. It consists of 79 items that can be synthetized into nine factors of the therapist’s emotional response to the patient: Overwhelmed/Disorganized, Helpless/Inadequate, Positive/Alliance, Special/Overinvolved, Sexualized, Disengaged, Parental/Protective, Criticized/Mistreated, Hostile/Angry. The present instrument has shown good previous levels of reliability coefficients for all of the subscales with Cronbach coefficients almost at or slightly above 0.80 (i.e. Helpless/Inadequate α = 0.90; Disengaged α = 0.78) [21].

## Data analyses

Normality assumption was verified for all quantitative variables. Correlations between variables were tested calculating Pearson coefficient or, if normality assumptions were violated, the nonparametric Spearman coefficient. More specifically with GSI score and TRQ factors was performed in order to assess any direct relationship between patient symptom severity and therapist response. A Pearson correlation with the SWAP-200 PD and Q scores and the TRQ factors was performed in order to explore the associations between the variables. Subsequently, only considering the variables that showed a significant correlation, a linear regression with enter method was applied [22]. More specifically multiple regression models were set with the single SWAP-200 PDs and Qs score as target variable and the single TRQ factor as independent determinant. A *p* < .001 Mahalanobi’s distance criterion was used to identify and skip multivariate outliers. All regression models were evaluated through statistically significant variation of R and Cohen’s [23] effect size f2. When regressions evidenced significant predictors, partial correlations were performed to exclude the influence of “patients’ age”, “patients’ years of study”, “number of hospitalizations”, “years of therapist experience” and “duration of the treatment” and “SCL-90-R GSI scores” (abbreviated in “Descriptive and clinical Variables” in the results and discussion section).

## Results

Table 1 shows information about patients, psychotherapists experience and the treatment duration.

**Table 1:** Patients and therapists characteristics and treatment information

	Patients age	Patients years of study	Hospitalization (N)	GSI scores	Therapists experience (years)	Treatments duration (months)
m	28.70	14.05	0.53	1.64	8.93	12.60
SD	7.99	3.66	5.05	0.55	11.85	9.61
Range	18–48	5–25	0–1	0.54–2.75	2–43	0–36

Descriptive statistics about patients’ personality traits and therapists’ emotional responses are reported in Tables 2 and 3 respectively. Helpless/Inadequate variable showed the highest scores indicating that this reaction was on average endorsed most strongly than others TRQ scores (i.e. positive/satisfying).

**Table 2:** Patients personality traits expressed in SWAP PD and Q scores descriptive statistics

	*M*	*SD*
*SWAP PD scores*		
Paranoid	46.62	8.29
Schizoid	45.08	7.36
Schizotypal	46.85	7.10
Antisocial	50.65	6.69
Borderline	58.27	9.84
Histrionic	55.22	8.92
Narcissistic	48.95	6.83
Avoidant	45.77	7.30
Dependent	50.70	7.00
Obsessive compulsive	40.74	9.07
High cunctioning	48.62	8.05
*SWAP Q scores*		
Dysphoric	*51.52*	7.26
Antisocial	50.75	6.49
Schizoid	45.73	7.25
Paranoid	47.20	7.80
Obsessive compulsive	44.23	8.66
Histrionic	55.35	10.05
Narcissistic	46.07	10.72
Avoidant	46.84	6.93
Depressive/high functioning	50.91	6.99
Emotionally dysregulated	55.53	8.74
Dependent	54.81	8.82
Hostility	47.44	8.58
High functioning	48.38	7.24

**Table 3:** Therapist Response Questionnaire (TRQ) descriptive statistics

TRQ scores	*M*	*SD*
Overwhelmed/disorganized	2.39	0.63
Helpless/inadequate	2.74	0.67
Positive/satisfying	2.39	0.61
Special/overinvolved	1.44	0.38
Sexualized	1.39	0.48
Disengaged	2.21	0.75
Parental/protective	2.16	0.60
Criticized/mistreated	2.09	0.62
Hostile/angry	2.22	0.68

No significant correlations were found between GSI score and TRQ factors (see Additional file 1 for details).

Correlations were computed to identify the SWAP-200 PD and Q scores that were statistically related (see Additional file 1 for details). Considering the significant results of the correlations, the regression analysis results identified the SWAP predictors that explained TRQ scores (Table 4).

**Table 4:** Regression analysis of changes in TRQ scores on SWAP PD and Q scores

Dependent variable	Independent variable	B	*t*	*P*
Overwhelmed/disorganized	PD antisocial	0.35	2.37	0.023°*
	PD obsessive compulsive	− 0.36	− 2.47	0.018°*
	Q dysphoric	0.37	2.55	0.015°*
	Q antisocial	0.37	2.57	0.014°*
	Q avoidant	− 0.32	− 2.16	0.037°*
Helpless/inadequate	PD obsessive compulsive	0.31	2.12	0.040*
	Q hostility	0.32	2.21	0.033°*
Positive satisfying	PD high functioning	0.25	1.69	0.099
	Q depressive/high functioning	0.37	2.52	0.016°*
Special/overinvolved	PD schizoid	− 0.42	− 2.94	0.005°**
	Q avoidant	− 0.39	− 2.75	0.009°**
Sexualized	P paranoid	− 0.37	− 2.54	0.01°**
	Q paranoid	− 0.35	− 2.42	0.03°*
Disengaged	PD schizoid	0.40	2.81	0.02°*
	PD avoidant	0.35	2.43	0.008°**
	PD obsessive compulsive	0.50	3.66	0.001°**
Parental/protective	Q dependent	0.31	2.08	0.04°*
	Q hostility	− 0.33	− 2.27	0.03°*
Criticized/mistreated	Q dysphoric	0.42	3.00	0.005°**
Hostile/angry	PD antisocial	0.33	2.23	0.031°*
	PD dependent	− 0.32	− 2.16	0.037°*
	Q antisocial	0.33	2.25	0.030°*

°Controlled for GSI scores, patients age, patients years of study, years of therapist experience and duration of the treatment

*p ≤ .05; **p ≤ .01;

More specifically, Overwhelmed/Disorganized TRQ scores were positively predicted by PD Antisocial (*R*^*2*^ = 0.121, *F*(1, 42) = 5.62, *p* ≤ .05), by Q Dysphoric (*R*^*2*^ = 0.137, *F*(1, 42) = 6.50, *p* ≤ .05) and by Q Antisocial (*R*^*2*^ = 0.138, *F*(1, 42) = 6.59, *p* ≤ .05) SWAP scores. Overwhelmed/Disorganized TRQ scores were negatively predicted by PD Obsessive compulsive (*R*^*2*^ = 0.130, *F*(1, 42) = 6.11, *p* ≤ .05) and Q Avoidant scores (*R*^*2*^ = 0.102, *F*(1, 42) = 4.67, *p* ≤ .037). We computed partial correlation to control for “Descriptive and clinical Variables.” All of the relations between SWAP variables and TRQ scores remained significant predictors (Partial correlation: PD Antisocial = 0.38, *p* = .009; Q Dysphoric = 0.39, *p* = .007; Q Antisocial = 0.40, *p* = .006; PD Obsessive compulsive = − 0.36, *p* = .012; Q Avoidant = − 0.36, *p* = .011) of Overwhelmed/disorganized TRQ scores.

Helpless/inadequate TRQ scores were positively predicted by PD Obsessive compulsive (*R*^*2*^ = 0.099, *F*(1, 42) = 4.50, *p* ≤ .05) and by Q Hostility (*R*^*2*^ = 0.106, *F*(1, 42) = 4.89, *p* ≤ .05) SWAP scores. However, when controlled for “Descriptive and clinical variables”, only Q Hostility (Partial correlation: 0.46 *p* = .002) remained a significant predictor.

Positive satisfying TRQ scores were not significantly predicted by PD High functioning (*R*^*2*^ = 0.065, *F*(1, 42) = 2.85, *p* = .099), but they were positively predicted by Q Depressive/high functioning scores (*R*^*2*^ = 0.134, *F*(1, 42) = 6.33, *p* ≤ .05) SWAP scores. Even when controlled for “Descriptive and clinical variables”, Q Depressive/high functioning remained a significant predictor (Partial Correlation = − 0.40, *p* = .006).

Special/overinvolved TRQ scores were negatively predicted by PD schizoid (*R*^*2*^ = 0.174, *F*(1, 42) = 8.65, *p* ≤ .05) and by Q Avoidant (*R*^*2*^ = 0.156, *F*(1, 42) = 7.58, *p* ≤ .01) SWAP scores. Even when controlled for “Descriptive and clinical variables”, both SWAP variables remained significant predictors (PD Schizoid = − 0.48, *p* = .001; Q Avoid = − 0.54, *p* = .000) of Special/overinvolved TRQ scores.

Sexualized TRQ Scores were negatively predicted by both P Paranoid (*R*^*2*^ = 0.136, *F*(1, 42) = 6.47, *p* ≤ .01) and Q Paranoid (*R*^*2*^ = 0.125, *F*(1, 42) = 5.84, *p* ≤ .05) SWAP scores. Even when controlled for “Descriptive and clinical variables”, P paranoid (Partial Correlation = − 0.309, *p* = .055) and Q paranoid remained a significant predictor (Partial Correlation = − 0.307, *p* = .029).

Disengaged TRQ Scores were positively predicted by PD Schizoid (*R*^*2*^ = 0.162, *F*(1, 42) = 7.91, *p* ≤ .01), PD Avoidant (*R*^*2*^ = 0.126, *F*(1, 42) = 5.89, *p* ≤ .05), PD Obsessive compulsive (*R*^*2*^ = 0.246, *F*(1, 42) = 13.41, *p* ≤ .01). Even when controlled for “Descriptive and clinical variables”, all these SWAP variables remained significant predictors (PD Schizoid Partial Correlation = 0.441, *p* = .002; PD Avoidant Partial Correlation = 0.371, *p* = .01; PD Obsessive compulsive Partial Correlation = 0.371, *p* = .01).

Parental Protective TRQ Scores were positively predicted by Q Dependent Scores (*R*^*2*^ = 0.096, *F*(1, 42) = 4.35, *p* ≤ .05), and negatively predicted by Q Hostility (*R*^*2*^ = 0.112, *F*(1, 42) = 5.16, *p* ≤ .05) SWAP scores. Even when controlled for “Descriptive and clinical variables”, all these SWAP variables remained significant predictors (Q dependent Partial Correlation = 0.370, *p* = .01; Q hostility Partial Correlation = 0.370, *p* = .01).

Criticized/ Mistreated TRQ Scores were positively predicted by Q Dysphoric SWAP scores (*R*^*2*^ = 0.180, *F*(1, 42) = 8.98, *p* ≤ .005). Even when controlled for “Descriptive and clinical variables”, Q Dysphoric scores remained a significant predictor (Partial Correlation = 0.453, *p* = .002).

Hostile/ angry TRQ Scores were positively predicted by PD Antisocial (*R*^*2*^ = 0.108, *F*(1, 42) = 4.98, *p* ≤ .05) and Q Antisocial (*R*^*2*^ = 0.110, *F*(1, 42) = 5.05, *p* ≤ .05) scores and negatively predicted by PD Dependent (*R*^*2*^ = 0.102, *F*(1, 42) = 4.66, *p* ≤ .05) scores. Even when controlled for “Descriptive and clinical variables”, all these SWAP variables remained significant predictors (PD Antisocial Partial Correlation = 0.471, *p* = .001; Q Antisocial Partial Correlation = 0.479, *p* = .001; PD Dependent Partial Correlation = − 0.313, *p* = .026).

## Discussion

The present paper provided important results related with therapist’s emotional reactions in mental facilities treatment specialised in PD. Our first aim was to investigate the direct relationship between patients’ symptom severity and therapist emotional response. Differently with previous data [8], we did not find significant relationships between the clinical severity of the symptoms and the therapist response. As expected, therapists working in these clinical settings may be less influenced by the clinical severity of patients. This may be related to the fact that therapists that work in such facilities have a specialised expertise in the treatment of PD [10].

Considering SWAP subscales, personality traits configurations showed significant correlations with specific TRQ scores. In line with the initial hypotheses, considering this kind of setting borderline traits were not related with strong emotional reaction in the therapists. This result is different previous results [5, 6], and may be due to the specific area of expertise of the psychotherapists that are used to work with patients with borderline traits. The specific facilities involved in the present study are focused on the treatment of borderline disorders and it may be that their therapists are able to work at the required emotional level with this kind of patients. Conversely, as different PDs are thought to have a different impact on therapy relationship, mostly based on their typical interpersonal schemas [9], therapists that works in the present facilities may have found more intense reactions working with less common schemas than usual. This may be true not only for A and C clusters that were related to negative therapist emotional responses, but also for other PDs related to B clusters a part from borderline. Similarly to Colli and colleagues findings [5], antisocial factors resulted correlated with hostile/angry therapist’s reactions and with overwhelmed/disorganized emotional responses. This is an interesting element showing that working with patients with antisocial traits can be related with intense anger and irritation even for therapists of this area of expertise. In a similar way narcissistic, and hostility/externalizing factors are related to criticized and mistreated emotional responses from the therapist.

A and C clusters were related with difficult-to-manage emotional responses in the therapists. More specifically, A cluster traits were positively associated with detached emotions and negatively related with proximity patterns such as involved or sexualized patterns. The schizoid factor was negatively correlated with special/overinvolved emotional response and positively correlated with disengaged responses, which is coherent with previous studies [5, 20].

In a similar way, C cluster Obsessive Compulsive and Avoidant traits were negatively related with Disengaged, Helpless/inadequate and Overwhelmed dimensions. In particular, the presence of obsessive-compulsive trait in different kinds of emotional response of the therapist is very interesting: the clinical interpretation of this data could lead us to highlight the difficult involvement in the treatment that characterizes this type of patient. Differently, considering the dependent/masochistic trait, the positive correlation with parental/protective emotional response of the therapist and the negative correlation with hostile angry emotions is confirmed [5].

The presence of positive and significant correlation between the psychological functioning of the patient [Q depressive (neurotic) high functioning] and the positive satisfying emotional response by the therapists is in line with previous findings [5]. Finally, the low level of sexualized emotional response of the therapist is a common factor that goes beyond personality traits. This is probably due to the public or institutional setting used for all the treatment. In our opinion, this could represent an important factor in the patient/therapist relationship.

The results of the present study confirm the influence of specific personality traits on the emotional response of the psychotherapist. Data showed that patient’s characteristics seem to have a great importance on therapist’ emotional responses compared to other variables such as age and educational level of the patient or years of therapist experience and duration of the treatment. This finding confirms the idea that personality characteristics and interpersonal functioning of patients is related with distinct emotional responses in therapists [8]. Differently from previous results [11], these relationships appear to be solid since only one case (Helpless/inadequate and PD Obsessive Compulsive) didn’t remain significant after controlling for the descriptive variables considered. This may be related to the fact that therapists considered in Liebman and Burnette paper [11] belonged to very different areas of expertise.

This study does not come without limitations. First, the same clinician provided data about both patients’ disorders and his or her own countertransference. Consequently the present results should be interpreted cautiously as they reflect the perception that the clinicians have about their patients and their emotional reactions as well. Although SWAP scales have previously showed high level of interrater reliability [17] and we controlled the results for setting variables (i.e. the duration of the treatment and the years of therapists experience), biases related to the ratings of different patients by the same therapists may also have occurred. A more rigorous research design, which should be conducted in future works, would include an independent assessment of patients’ personality disorders or the use of an observer-rated analysis of therapists’ reactions, or both. The present exploratory study provided correlations between 24 SWAP variables and 9 TRQ reactions. This multiple testing may lead to Type 1 error i.e. false positive, future confirmatory study may therefore verify these results with more refined analyzes. Finally, the sample is representative of patients with severe mental disorders, but the limited number of patients prevents us from more general conclusions. Even if the present study was proposed to all mental facilities’ patients not everyone accepted. It’s possible that those who did not accept may show recursive configurations in terms of personality traits and psychological functioning that may be worthy of interest.

## Conclusions

Despite some limitation, this work confirms the value of therapists’ emotional response as a useful tool in understanding psychological processes related to clinical practice focused on patients with severe mental disorders. Moreover, the present paper evidenced that most of the significance considering the effects of patients and therapists variables related to a very specific clinical setting. Even when controlled for clinical variable related to a very specific clinical setting (severity condition, duration of the treatment, patients’ age, educational level of the patient and years of therapist experience), most of SWAP-200 traits appeared to be significant predictors of therapist’ emotional responses. This result stresses the need to take in high consideration the features of the psychotherapist. As the results of the Third Interdivisional APA Task Force on Evidence-Based Relationships and Responsiveness showed [24], each psychotherapeutic treatment presents more possibilities to reach a good outcome if the psychotherapist will be able to tailor his approach and his personality features in relation to personality, culture, and preferences of the patient. For every therapist, handling one’s own emotional responses is a crucial aspect to provide effective and balanced treatments. As in other European countries [25], sharing simple, principle-driven, ‘common-factors’ framework for the treatment of PDs, both in and outside of Italian specialized settings could be a relevant issue. Future research could assess the effectiveness of the PD treatments based on common factors that can integrate the knowledge of the scientific community and professional expertise.

The present findings suggest that when only facilities specialised in personality disorders’ treatments are involved, the relationship between patient personality characteristics and emotional response in therapists seem to be not influenced by the clinical severity of the patient. The present reactions, and therefore the patient-therapist relationship could be particularly context-dependent and may be influenced by the therapist area of expertise, which is an aspect with both clinical and scientific implications.

## Supplementary Information

**Additional file 1.** Supplementary material.

## Data Availability

The datasets used and analysed during the current study cannot be made publicly available due to IRB restrictions, but may be available from the corresponding author on reasonable request.
