# Supplementary material for: Psychotherapists’ emotional reactions to patients’ personality trait in personality disorder treatment settings: an exploratory study
Source: BMC Psychol. 2021 May 6;9:74. doi: 10.1186/s40359-021-00580-z (PMC8103645; doi:10.1186/s40359-021-00580-z)
Supplement: Supplementary file 1 — Additional file 1. Supplementary material. [file 40359_2021_580_MOESM1_ESM.docx]

**Table S1**

*Correlation coefficients between Global Severity Index (GSI) from SCL-90R and TRQ factors.*

|  | *GSI (1)* |
| --- | --- |
| *Overwhelmed/disorganized (2)* | .24 |
| *Helpless/inadequate (3)* | -.01 |
| *Positive/satisfying (4)* | -.19 |
| *Special/overinvolved (5)* | -.06° |
| *Sexualized (6)* | -.07° |
| *Disengaged (7)* | -.09 |
| *Parental/protective (8)* | -.11 |
| *Criticized/mistreated (9)* | -.00° |
| *Hostile/angry (10)* | -.11 |

° Spearman rank correlational coefficient

**Table S2**

*Correlations coefficients between SWAP PD and Q scores and TRQ factors.*

| *SWAP PD Scores* | *Overwhelmed/*  *disorganized* | *Helpless/*  *inadequate* | *Positive/*  *satisfying* | *Special/*  *overinvolved* | *Sexualized* | *Disengaged* | *Parental/*  *protective* | *Criticized/*  *mistreated* | *Hostile/ angry* |
| --- | --- | --- | --- | --- | --- | --- | --- | --- | --- |
| *Paranoid* | .17 | .15 | .01 | -.05° | -.38*° | -.12 | -.18 | .04° | .09 |
| *Schizoid* | -.18° | .22° | -.19° | -.36*° | .05° | .40**° | -.21° | .22° | .04° |
| *Schizotypal* | -.06 | .14 | -.17 | -.20° | -.23° | .14 | -.12 | .05° | -.03 |
| *Antisocial* | .35* | .09 | -.15 | .03° | -.09° | -.13 | .02 | .07° | .33* |
| *Borderline* | .18° | -.22° | .06° | .10° | -.29° | -.36*° | .18° | -.09° | -.22° |
| *Histrionic* | .29 | -.13 | -.09 | .13° | -.06° | -.24 | .15 | -.12° | -.07 |
| *Narcissistic* | .26 | .18 | -.15 | .05° | -.04° | .05 | -.16 | .14° | .28 |
| *Avoidant* | -.25 | .18 | -.08 | -.26° | .04° | .35* | -.13 | .25° | -.01 |
| *Dependent* | -.21 | -.15 | .03 | -.06° | .02° | .09 | .11 | -.03° | -.32* |
| *Obsessive compulsive* | -.31*° | .36*° | -.11° | -.29° | .18° | .53** | -.29° | .20° | .17° |
| *High Functioning* | -.25° | -.15° | .29° | .07° | .10° | .04° | -.06° | -.09° | -.09° |
| *SWAP Q Scores* |  |  |  |  |  |  |  |  |  |
| *Dysphoric* | .37* | .23 | .01 | .18° | .20° | .02 | .18 | .44**° | .19 |
| *Antisocial* | .37* | .13 | -.13 | .01° | -.09° | -.14 | -.01 | .15° | .33* |
| *Schizoid* | -.15 | .20 | -.26 | -.28° | -.15° | .28 | -.21 | .09° | .01 |
| *Paranoid* | .13 | .05 | .11 | -.03° | -.37*° | -.26 | -.12 | .02° | .02 |
| *Obsessive compulsive* | -.22° | .02° | .28° | .06° | .10° | .13° | -.11° | -.05° | -.01° |
| *Histrionic* | .13 | -.29 | .05 | .27° | -.06° | -.25 | .22 | -.17° | -.23 |
| *Narcissistic* | .03 | .16 | -.23 | -.14 | .03° | .22 | -.20 | .19° | .21 |
| *Avoidant* | -.32* | .11 | -.07 | -.37* | .09° | .39** | -.16 | .20° | -.08 |
| *Depressive /  high functioning* | -.21 | -.17 | .37* | .23° | .07° | .00 | .04 | -.04° | -.18 |
| *Emotionally Dysregulated* | .21 | .01 | -.04 | -.01° | -.24° | -.21 | .05 | .08° | -.04 |
| *Dependent* | .11 | -.28 | -.04 | .13° | .07° | -.19 | .31* | -.11° | -.20 |
| *Hostility* | .10° | .33*° | -.16° | -.17° | -.11° | .24° | -.29* | .29° | .23° |
| *High Functioning* | -.19° | -.17° | .30*° | .11° | -.00° | -.06° | -.09° | -.13° | -.18° |

* p ≤ .05; ** p ≤ .01; ° Spearman rank correlational coefficient
